# Effectiveness of oral versus long-acting antipsychotic treatment early-phase schizophrenia patients: an open-label randomized trial

**DOI:** 10.1192/j.eurpsy.2022.356

**Published:** 2022-09-01

**Authors:** I. Winter, M. Davidson, W. Fleischhacker, R. Kahn

**Affiliations:** 1 University Medical Center Utrecht, Psychiatry, Utrecht, Netherlands; 2 Minerva Neurosciences, Psychiatry, Waltham, United States of America; 3 University of Innsbruck, Psychiatry, Innsbruck, Austria; 4 Mount Sinai, Psychiatry, New York, United States of America

**Keywords:** long-acting antipsychotics, oral antipsychotic, all-cause discontinuation, schizophrénia

## Abstract

**Introduction:**

Schizophrenia is a chronic psychiatric illness with periods of remission and relapse. Patients vary in the frequency and severity of relapse, time until relapse and time in remission. Discontinuation of antipsychotic medication is by far the most important reason for relapse. A possible method to optimize medication adherence is to treat patients with long-term, depot medication rather than oral medication.

**Objectives:**

Primary objective is to compare all cause discontinuation rates in patients with schizophrenia randomized to either one of the two depot medications (aripiprazole depot or paliperidone palmitate) with patients randomized to either one of the two oral formulations of the same medication (aripiprazole or paliperidone) over an 19 month follow-up period.

**Methods:**

Pragmatic, randomized, open label, multicenter, multinational comparative trial consisting of a 19 month treatment period. Patients aged 18 years or older, having experienced the first psychosis 1-7 years ago, currently meeting DSM-IV-R criteria for schizophrenia. Patients are randomized 1:1:1:1 to paliperidone palmitate, aripiprazole depot, oral aripiprazole or oral paliperidone. The primary outcome is all cause discontinuation.

**Results:**

In the Intent to Treat sample (n=511), no difference was found in time to ACD between the combined oral and combined depot treatment arms, nor between the four individual treatment arms.

**Conclusions:**

Even though the scientific evidence comparing oral and depot medication has been inconsistent, most studies were conducted in rigorous clinical settings, which may have biased those results. In contract, given the pragmatic, open label design of the current trial, the results may be more representative of common daily practice.

**Disclosure:**

No significant relationships.

